# Non-Work-Matched HIIT and MIIT Partially Restore Exerkine-Related and Mitochondrial Gene Expression in Diabetic Rat Skeletal Muscle

**DOI:** 10.3390/biom16081152

**Published:** 2026-08-07

**Authors:** Saeed Rezae, Payam Abasian Mehr, Parisa Pournemati, Ismail Laher, Özgür Eken, Monira I. Aldhahi

**Affiliations:** 1 Department of Exercise Physiology, Faculty of Physical Education and Sport Sciences, Ferdowsi University of Mashhad, Mashhad 9177948974, Iran; saeed.rezaee@mail.um.ac.ir (S.R.); payam.abasianmehr@mail.um.ac.ir (P.A.M.); 2 Department of Exercise Physiology, Faculty of Physical Education and Sport Sciences, University of Tehran, Tehran 1417935840, Iran; pournemati@ut.ac.ir; 3 Department of Anesthesiology, Pharmacology and Therapeutics, Faculty of Medicine, The University of British Columbia, Vancouver, BC V6T 1Z3, Canada; ismail.laher@ubc.ca; 4 Department of Physical Education and Sport Teaching, Faculty of Sports Sciences, Inonu University, Malatya 44280, Türkiye; ozgur.eken@inonu.edu.tr; 5 Department of Rehabilitation Sciences, College of Health and Rehabilitation Sciences, Princess Nourah bint Abdulrahman University, Riyadh 11671, Saudi Arabia

**Keywords:** exerkines, interval training, skeletal muscle, mitochondrial biogenesis, type 2 diabetes, FNDC5, osteocrin

## Abstract

Skeletal muscle mitochondrial dysfunction and altered myokine signaling contribute to insulin resistance in type 2 diabetes. This study compared the effects of non-work-matched high-intensity interval training (HIIT) and moderate-intensity interval training (MIIT) on skeletal muscle exerkine/myokine- and mitochondrial biogenesis-related gene expression and systemic metabolic indices in streptozotocin-nicotinamide-induced diabetic rats. Twenty-four male Wistar rats were initially allocated to healthy control, diabetic control, MIIT, or HIIT groups; after predefined treadmill-familiarization exclusions, five animals per group were analyzed. Training was performed for 6 weeks, three sessions per week, with MIIT prescribed at 70% maximal aerobic speed and HIIT at 90% maximal aerobic speed. Gastrocnemius expression of FNDC5, OSTN, PGC-1α, TFAM, CCO, and UCP3 was quantified by RT-qPCR, and fasting glucose, insulin, lipid variables, HOMA-IR, HOMA-β, QUICKI, and TyG index were assessed. Diabetes reduced all targeted transcripts and impaired insulin-related metabolic indices. Both MIIT and HIIT partially restored myokine- and mitochondrial-related transcripts compared with diabetic controls, with no significant differences between training protocols for most molecular outcomes. HIIT produced lower fasting insulin and HOMA-IR than MIIT but imposed a greater estimated cumulative workload. These findings indicate that interval training partly attenuates diabetes-associated transcriptional and insulin-related metabolic disturbances, while intensity-specific conclusions require work-matched designs.

## 1. Introduction

Type 2 diabetes (T2D) is a complex metabolic disorder characterized by insulin resistance, impaired glycaemic regulation, altered lipid metabolism, and progressive disruption of skeletal muscle energy homeostasis [1,2]. Although pharmacological therapy remains central to diabetes management, adverse effects, variable treatment responses, and incomplete metabolic control highlight the need for adjunctive non-pharmacological strategies [3,4]. Exercise training is therefore widely recommended as a core component of T2D prevention and management because it can improve insulin sensitivity, cardiorespiratory fitness, and skeletal muscle metabolic function [5,6,7].

Skeletal muscle is a major site of insulin-stimulated glucose disposal, and mitochondrial dysfunction in this tissue is closely linked to insulin resistance and impaired metabolic flexibility [1,8,9]. Exercise-induced mitochondrial adaptation is commonly regulated through transcriptional networks involving peroxisome proliferator-activated receptor gamma coactivator 1-alpha (PGC-1α) and downstream targets such as mitochondrial transcription factor A (TFAM), cytochrome c oxidase (CCO), and uncoupling protein 3 (UCP3), which are related to mitochondrial transcription, oxidative metabolism, and energy regulation [10,11,12]. Skeletal muscle also acts as an endocrine organ through the expression and secretion of myokines. Exercise-responsive factors such as FNDC5, which encodes irisin, and OSTN, also known as osteocrin or musclin, have been implicated in glucose metabolism, mitochondrial regulation, and skeletal muscle adaptation [11,13].

Interval training has attracted attention because it may induce robust metabolic adaptations within a relatively short training time [5,6,12]. HIIT typically combines brief periods of vigorous exercise with recovery intervals, whereas MIIT uses lower-intensity interval work. Previous studies in diabetic rodent models suggest that interval-training protocols can improve metabolic control and modulate skeletal muscle molecular pathways [14,15,16]. However, many studies have focused on either systemic metabolic outcomes or a limited number of mitochondrial markers, and fewer have simultaneously examined myokine-related transcripts and a broader mitochondrial biogenesis-related gene panel. In addition, comparisons between HIIT and lower-intensity interval protocols are often difficult to interpret when total external workload is not matched [14,17].

Accordingly, the present study investigated the effects of 6 weeks of HIIT and MIIT on gastrocnemius expression of FNDC5, OSTN, PGC-1α, TFAM, CCO, and UCP3, together with fasting glucose, insulin, lipid variables, HOMA-IR, HOMA-β, QUICKI, and TyG index in an STZ–NA rat model of T2D. Because the protocols were matched for session duration and interval structure but not for total external workload, between-protocol differences were interpreted as reflecting the combined influence of relative exercise intensity and accumulated running load. We hypothesized that both MIIT and HIIT would partially restore diabetes-associated reductions in skeletal muscle myokine- and mitochondrial-related gene expression compared with diabetic controls, and that HIIT would produce greater improvement in insulin resistance than MIIT under these non-work-matched conditions.

## 2. Materials and Methods

### 2.1. Experimental Design and Animal Model

This randomized controlled animal experiment used 24 male Wistar rats (initial body mass: 180–190 g) obtained from the Pasteur Institute of Iran. The animals were housed under controlled laboratory conditions (temperature 23–25 °C, humidity 25–30%, 12:12 h light–dark cycle, lights on at 07:00) with ad libitum access to standard chow and water. Rats were group-housed three per cage in standard polypropylene cages, and cage allocation was balanced across groups to reduce potential cage-related effects. The individual animal was considered the experimental unit for all analyses.

After a one-week acclimatization period at the animal facility of the Faculty of Sport Sciences, University of Tehran, animals were randomly assigned using a computer-generated sequence to one of four groups, as follows: HC, DC, MIIT, or HIIT (*n* = 6 per group). Type 2 diabetes was subsequently induced in the DC, MIIT, and HIIT groups using the STZ–NA protocol described in Section 2.4. Diabetes status was confirmed using predefined glycemic thresholds, and baseline fasting glucose was recorded before initiation of the exercise intervention to confirm metabolic comparability among diabetic groups.

Inclusion and exclusion criteria were established a priori. Animals were required to successfully complete treadmill familiarization and demonstrate the ability to sustain running during the adaptation phase. During treadmill adaptation, one animal from each group failed to meet these criteria and was excluded prior to the initiation of the training intervention. Consequently, the final analyzed sample consisted of five animals per group (*n* = 5/group; total *n* = 20). No animals were excluded after the start of the formal intervention or during outcome assessment.

Animal welfare was monitored daily throughout the study. Clinical signs including body weight loss, grooming behavior, mobility, and general activity were assessed to identify potential distress. Humane endpoints were predefined; animals exhibiting severe lethargy, inability to ambulate, or weight loss exceeding 20% were to be removed from the study. No animals reached humane endpoint criteria during the experimental period.

All experimental procedures were approved by the Research Ethics Committee of the Sport Sciences Research Institute (Approval ID: IR.SSRC.REC.1402.162; approval date: 20 September 2023) and were conducted in accordance with institutional and national guidelines for animal care and experimentation.

### 2.2. Sample Size and Power Analysis

Sample size was estimated a priori using G*Power software, version 3.1.9.7 (Heinrich Heine University Düsseldorf, Düsseldorf, Germany)**,** for a one-way fixed-effects ANOVA with four groups (alpha = 0.05, power = 0.80). Although HOMA-IR was defined as the primary metabolic outcome, the power calculation was based on FNDC5 expression because directly comparable prior effect-size estimates for HOMA-IR under the present experimental conditions were limited [18,19,20]. A large expected effect size (f = 0.70) indicated that six animals per group would be sufficient. After predefined exclusion during treadmill familiarization, the final analyzed sample consisted of five animals per group. Thus, the study was adequately powered to detect large effects, whereas small-to-moderate effects and HIIT-versus-MIIT differences should be interpreted cautiously.

### 2.3. Randomization and Blinding

Randomization was performed after acclimatization using a computer-generated sequence produced with Research Randomizer, version 4.0 (www.randomizer.org; Urbaniak and Plous, web-based software), by an investigator not involved in outcome assessment. Investigators responsible for RT-qPCR and biochemical assays were blinded to group allocation, and samples were labeled with anonymous codes until analyses were complete. Blinding of personnel supervising treadmill exercise was not feasible because of the behavioral nature of the intervention; however, laboratory outcome assessment remained blinded.

### 2.4. Induction of Diabetes

Diabetes was induced using the streptozotocin–nicotinamide (STZ–NA) model, a well-established method for producing a type-2-diabetes-like phenotype in rodents. Following a 12 h overnight fasting period, the rats received an intraperitoneal injection of streptozotocin (STZ; 65 mg/kg; Sigma-Aldrich, St. Louis, MO, USA), freshly dissolved in 0.1 M citrate buffer (pH 4.5). Fifteen minutes later, nicotinamide (NA; 120 mg/kg) was administered intraperitoneally [21,22,23]. Blood glucose was measured from a tail-vein sample 72 h after injection under fasting conditions using a calibrated glucometer (IME-DC GmbH, Hof, Germany). Rats with fasting blood glucose ≥126 mg/dL were considered to have disrupted glucose homeostasis and were provisionally classified as diabetic [21,22,23].

To confirm stable hyperglycemia, fasting blood glucose was reassessed one week after induction. Animals with fasting blood glucose ≥200 mg/dL at this time point were retained in the diabetic groups, whereas animals not reaching this threshold were excluded from further analysis. Baseline fasting blood glucose values were recorded before the initiation of the exercise intervention to ensure metabolic comparability among the experimental groups.

### 2.5. Treadmill Adaptation, Maximal Aerobic Speed (MAS) Determination, and Interval Training Protocol

**Figure 1:**
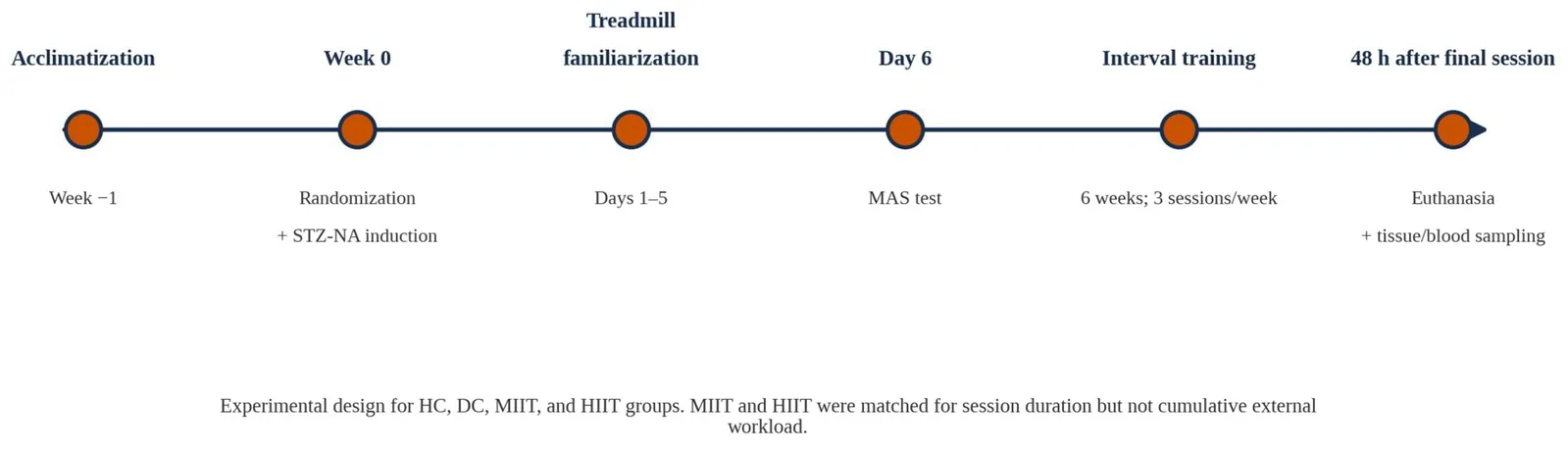
Experimental design and timeline of the study, including diabetes induction, treadmill adaptation, maximal aerobic speed (MAS) assessment, and the six-week interval training intervention.

Twenty-four hours after diabetes induction, the rats underwent five consecutive days of treadmill familiarization at 0° incline. The animals ran for 50 min per session, beginning at 5 m·min^−1^ on day 1 and increasing by 1 m·min^−1^ each day to minimize stress and habituate the animals to the apparatus [24,25]. On day 6, maximal aerobic speed (MAS) was operationally determined using a graded incremental treadmill test beginning at 5 m·min^−1^, with speed increased by 1 m·min^−1^ every minute until voluntary exhaustion. MAS was defined as the highest treadmill speed reached at voluntary exhaustion. Respiratory gas exchange (VO_2_ and CO_2_) was not measured because the treadmill was not equipped with a hermetic metabolic chamber; therefore, MAS should be interpreted as a performance-derived maximal running speed rather than a gas-exchange-verified speed at VO_2_max. Exercise training began 48 h after MAS determination and was performed three sessions per week for 6 weeks. Each training session consisted of a 5 min warm-up at 5 m·min^−1^, a 40-min interval block, and a 5 min cool-down at 5 m·min^−1^. MIIT alternated 1 min at 70% MAS with 1 min at 50% MAS for 20 cycles, whereas HIIT alternated 1 min at 90% MAS with 1 min at 50% MAS for 20 cycles. The control animals were placed on the treadmill for the same duration without running to control for handling and environmental exposure. MAS was reassessed during Weeks 1, 3, and 6, and training speeds were updated to maintain the prescribed relative intensities. The experimental sequence is presented in Figure 1, and the protocol characteristics are summarized in Table 1.

#### External Workload Calculation

Session duration, interval structure, treadmill incline, and weekly training frequency were identical between MIIT and HIIT. However, because work-interval intensity was prescribed at 70% versus 90% MAS, the protocols were not matched for external workload. Estimated session distance was calculated as MIIT = 50 + 24 × MAS and HIIT = 50 + 28 × MAS, where MAS is expressed in m·min^−1^. Based on group-mean MAS values at Weeks 1, 3, and 6 and three two-week phases of training, cumulative running distance was estimated as 13,457 m for MIIT and 16,692 m for HIIT, indicating approximately 24.0% greater external workload in the HIIT group. Therefore, the present design compares two practically applied interval-training prescriptions rather than isolating exercise intensity independently of accumulated mechanical work.

### 2.6. Tissue Collection and Biochemical Analysis

#### 2.6.1. Pre-Analytical Standardization and Euthanasia

The animals were euthanized 48 h after the final training session following a 12 h overnight fast to minimize acute exercise-induced perturbations and standardize metabolic conditions [26,27,28]. General anesthesia was induced via intraperitoneal injection of ketamine (80 mg·kg^−1^) and xylazine (10 mg·kg^−1^) [28,29]. Adequate anesthetic depth was confirmed by the absence of pedal withdrawal and corneal reflexes prior to euthanasia. The animals were subsequently euthanized by decapitation in accordance with institutional ethical guidelines. All procedures were performed at a consistent time of day (±1 h) to control for circadian variation.

#### 2.6.2. Skeletal Muscle Sampling and Processing

Immediately following euthanasia, the gastrocnemius muscle (combined medial and lateral heads) was rapidly excised, cleared of connective tissue, snap-frozen in liquid nitrogen within <60 s, and stored at −80 °C until analysis. The gastrocnemius was selected because it is a large locomotor muscle recruited during treadmill running, provides sufficient tissue for standardized RNA analysis, and has a heterogeneous but predominantly fast-twitch fiber profile; the soleus is predominantly slow oxidative and represents a different muscle phenotype. Whole-muscle sampling was used to capture the integrated transcriptional response of the gastrocnemius. Tissue handling time was standardized across all animals to minimize RNA degradation and pre-analytical variability.

#### 2.6.3. Blood Collection and Plasma Preparation

Trunk blood was collected immediately after decapitation into pre-chilled EDTA-coated tubes and processed within 15 min. The seamples were centrifuged at 1800× *g* for 15 min at 4 °C. Plasma was separated, aliquoted into low-binding microtubes, and stored at −80 °C until biochemical analysis. To prevent degradation, repeated freeze–thaw cycles were avoided by using single-use aliquots.

#### 2.6.4. Biochemical Assays

Fasting plasma glucose concentrations (mg·dL^−1^) were determined enzymatically using an automated analyzer (Alpha Classic Auto-Analyzer, Tehran, Iran) based on the glucose oxidase–peroxidase (GOD-PAP) method. Plasma insulin concentrations (mIU·L^−1^) were quantified using a rat-specific enzyme-linked immunosorbent assay (ELISA) kit (RayBiotech, Peachtree Corners, GA, USA) according to the manufacturer’s instructions. Absorbance was measured at 450 nm, and concentrations were calculated using a four-parameter logistic (4-PL) standard curve.

Plasma lipid parameters were assessed enzymatically. Total cholesterol and triglycerides (mg·dL^−1^) were measured using commercial kits based on CHOD-PAP and GPO-PAP methods, respectively, at 37 °C following the manufacturer’s protocols. All biochemical measurements were performed in duplicate, and the mean values were used for statistical analysis.

#### 2.6.5. Derived Metabolic Indices

To comprehensively evaluate metabolic status, the following indices were calculated from fasting plasma values using standard equations consistent with the measurement units applied in the present study [30,31,32,33]:

HOMA-IR = [Insulin (mIU·L^−1^) × Glucose (mg·dL^−1^)]/405

QUICKI = 1/[log_10_ Insulin (mIU·L^−1^) + log_10_ Glucose (mg·dL^−1^)]

HOMA-β = [360 × Insulin (mIU·L^−1^)]/[Glucose (mg·dL^−1^) − 63]

TyG index = ln [Triglycerides (mg·dL^−1^) × Glucose (mg·dL^−1^)/2]

Higher HOMA-IR and TyG values indicate greater insulin resistance, whereas higher QUICKI values indicate greater insulin sensitivity. HOMA-β was used as a surrogate index of β-cell functional capacity under fasting conditions.

### 2.7. Quantitative Real-Time PCR (RT-qPCR) Analysis

Total RNA was isolated from gastrocnemius muscle samples using QIAzol Lysis Reagent (Qiagen, Hilden, Germany) according to the manufacturer’s instructions. RNA concentration and purity were assessed using a NanoDrop 2000 spectrophotometer, and samples with A260/A280 ratios of 1.8–2.1 and A260/A230 ratios ≥1.8 were accepted. DNase-treated RNA was reverse transcribed using the QuantiTect Reverse Transcription Kit (Qiagen, Hilden, Germany). RT-qPCR was performed using an ABI StepOne Real-Time PCR System with SYBR Green chemistry. Technical duplicates were used for all reactions, no-template controls were included, and melt-curve analysis confirmed amplification specificity. Relative expression was quantified using the 2^−ΔCt^ method with GAPDH as the reference gene. Statistical analyses were performed on ΔCt values to reduce distributional skewness, whereas 2^−ΔCt^ values are presented for interpretability. Primer efficiencies ranged from 95% to 105% with R2 ≥ 0.99. Primer sequences and accession numbers are presented in Table 2.

FNDC5 and OSTN are reported using the target nomenclature applied in the present assay; accession numbers are provided for precise identification. CCO refers to the cytochrome c oxidase target listed in this table. GAPDH was used as the reference gene.

### 2.8. Statistical Analysis

Data are presented as mean ± SD unless otherwise specified. The individual animal was used as the experimental unit. Normality of model residuals was evaluated using Shapiro–Wilk tests and Q-Q plots, and homogeneity of variance was assessed using Levene’s test. Because each group contained five animals, the limited power of formal normality and variance tests was explicitly recognized. Parametric analyses were retained because the design was balanced, and visual residual diagnostics did not indicate major departures from normality or materially unequal variances. In balanced designs, ANOVA is comparatively robust to moderate distributional departures; nevertheless, results were interpreted together with effect sizes, 95% confidence intervals, and exact *p* values, and non-significant findings were not treated as evidence of equivalence. Endpoint molecular and metabolic outcomes were analyzed using one-way ANOVA. When the omnibus test was significant, Tukey’s HSD test was used for post hoc pairwise comparisons. Gene-expression outcomes were analyzed using ΔCt values, while 2^−ΔCt^ values are shown for interpretability. To control for multiple testing across gene-expression outcomes, Benjamini–Hochberg false discovery rate correction was applied to omnibus ANOVA *p*-values. Body weight and MAS were analyzed using mixed-design repeated-measures ANOVA with time as the within-subject factor and group as the between-subject factor. Mauchly’s test was used to assess sphericity, with Greenhouse–Geisser correction when required. Effect sizes were reported as eta squared (η^2^) for one-way ANOVA and partial eta squared for repeated-measures models. Mean differences are reported with 95% confidence intervals where applicable. Exploratory correlations were interpreted as associative rather than causal. Statistical significance was set at *p* < 0.05.

## 3. Results

### 3.1. Body Weight Changes During the Nine-Week Experiment

**Figure 2:**
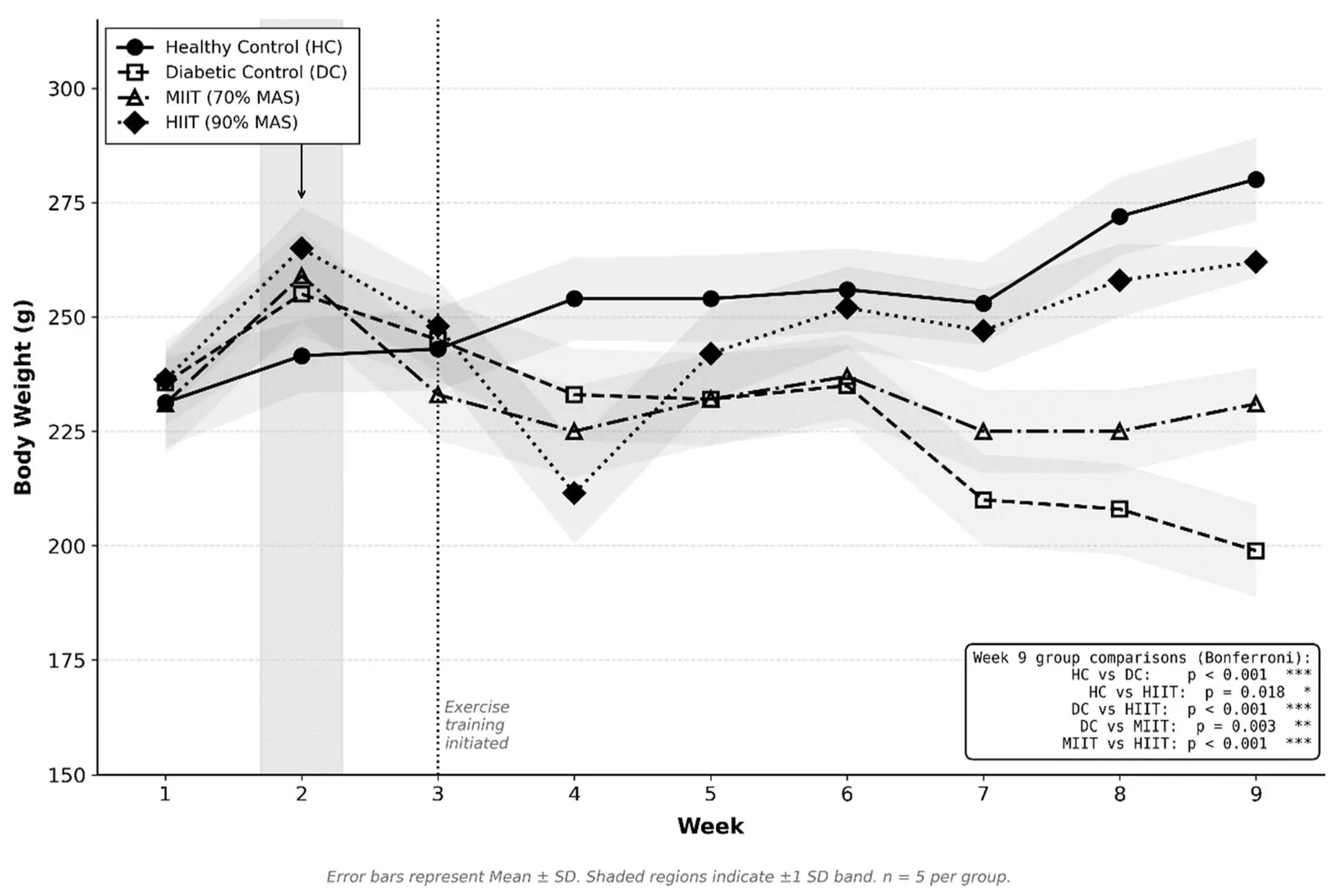
Body weight trajectories across the nine-week experimental period. Values represent Mean ± SD (*n* = 5 per group). Shaded regions indicate ±1 SD band. The grey bar at Week 2 indicates STZ–NA administration. The vertical dotted line at Week 3 marks the initiation of exercise training. Week 9 pairwise comparisons are Tukey-adjusted pairwise comparisons. HC = Healthy Control; DC = Diabetic Control; MIIT = Moderate-Intensity Interval Training (70% MAS); HIIT = High-Intensity Interval Training (90% MAS). * *p* < 0.05; ** *p* < 0.01; *** *p* < 0.001.

Body weight did not differ between groups at baseline (all *p* > 0.05), confirming successful matching prior to intervention. Following diabetes induction, divergent trajectories emerged across groups (Figure 2).

Mixed-design repeated-measures ANOVA showed significant effects of time (F(2.15, 34.40) = 94.30, *p* < 0.001, partial η^2^ = 0.94), group (F(3, 16) = 35.69, *p* < 0.001, η^2^ = 0.87), and time × group interaction (F(6.45, 34.40) = 12.10, *p* < 0.001), indicating different body-weight trajectories across the experimental period.

At Week 9, a large between-group effect was observed (F (3, 16) = 101.20, *p* < 0.001, η^2^ = 0.95). The DC group had lower body weight than the HC, whereas both training groups showed higher body weight than the DC. HIIT was also higher than MIIT at Week 9. These findings indicate that interval training attenuated diabetes-associated body-weight loss, with the greatest preservation observed in the HIIT group under the present non-work-matched conditions.

### 3.2. Changes in Maximal Aerobic Speed

**Figure 3:**
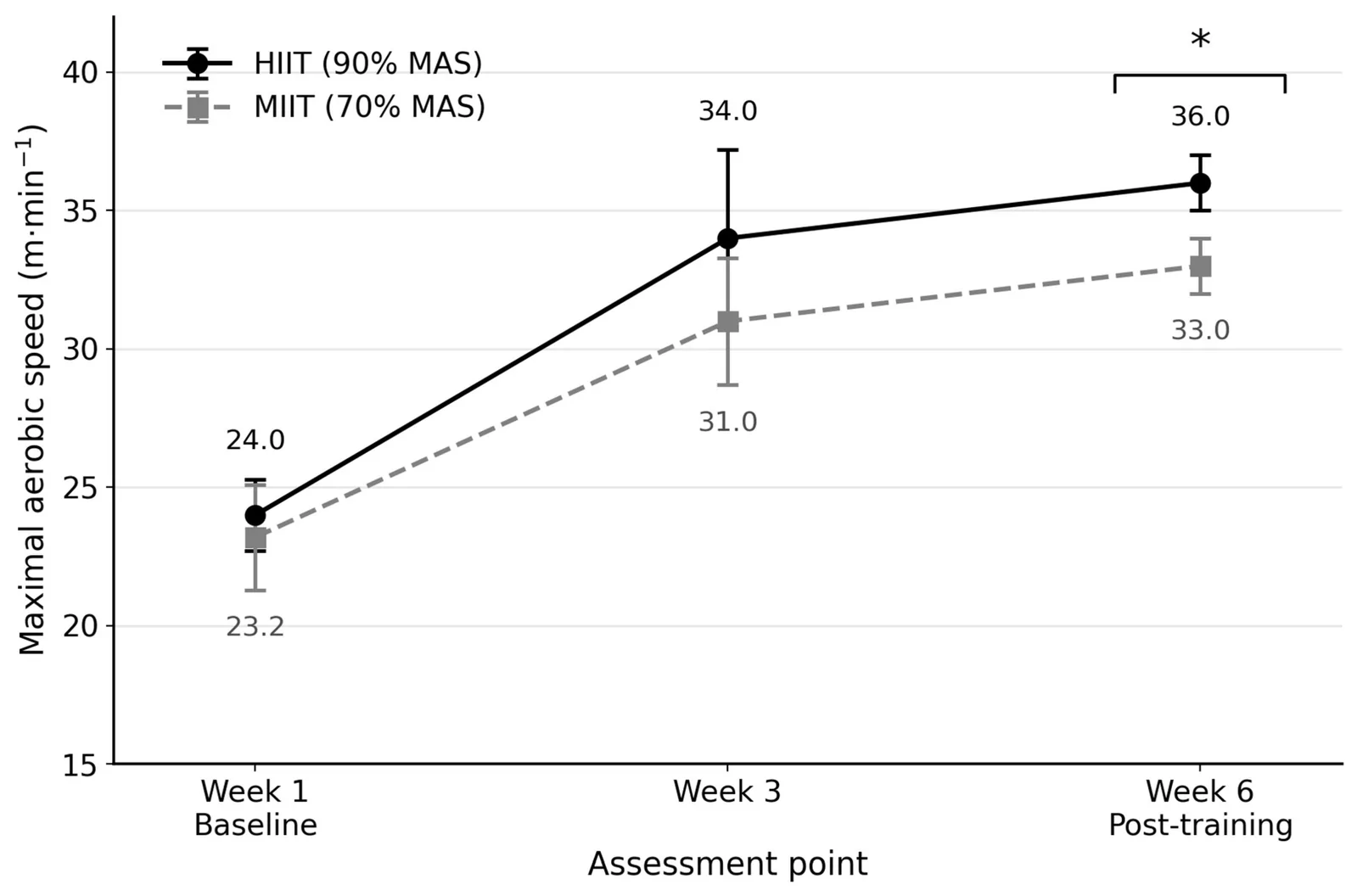
Changes in maximal aerobic speed (MAS) across three assessment points (Weeks 1, 3, and 6) in diabetic rats undergoing high-intensity interval training (HIIT; 90% MAS) or moderate-intensity interval training (MIIT; 70% MAS). Symbols with error bars denote group means ± SD (*n* = 5 per group). The significance bracket indicates a between-group difference at Week 6 (* *p* < 0.05); groups did not differ significantly at baseline or Week 3. MAS, maximal aerobic speed.

To characterize the functional aerobic response to the two interval-training protocols, MAS was assessed at baseline (Week 1), mid-intervention (Week 3), and after six weeks of training (Week 6). Baseline MAS did not differ significantly between HIIT and MIIT (24.00 ± 1.58 vs. 23.20 ± 1.92 m·min^−1^, respectively; *p* > 0.05). Both groups improved across time, with MAS reaching 36.00 ± 0.71 m·min^−1^ in HIIT and 33.00 ± 0.71 m·min^−1^ in MIIT at Week 6. Mixed repeated-measures ANOVA showed a significant main effect of time and a significant group × time interaction, indicating progressive aerobic adaptation in both training groups and a greater increase in HIIT. The main effect of group was not significant. Exact values are displayed in Figure 3.

### 3.3. Estimated External Workload

Estimated running distance increased across the intervention in both training groups because training speeds were updated according to MAS. Based on the group-mean MAS values at Weeks 1, 3, and 6, cumulative running distance over 18 sessions was greater in HIIT than MIIT (16,692 m vs. 13,457 m), corresponding to approximately 24.0% higher external workload. Therefore, subsequent between-protocol comparisons should be interpreted as reflecting the combined effects of higher relative intensity and greater accumulated workload rather than exercise intensity alone.

**Figure 4:**
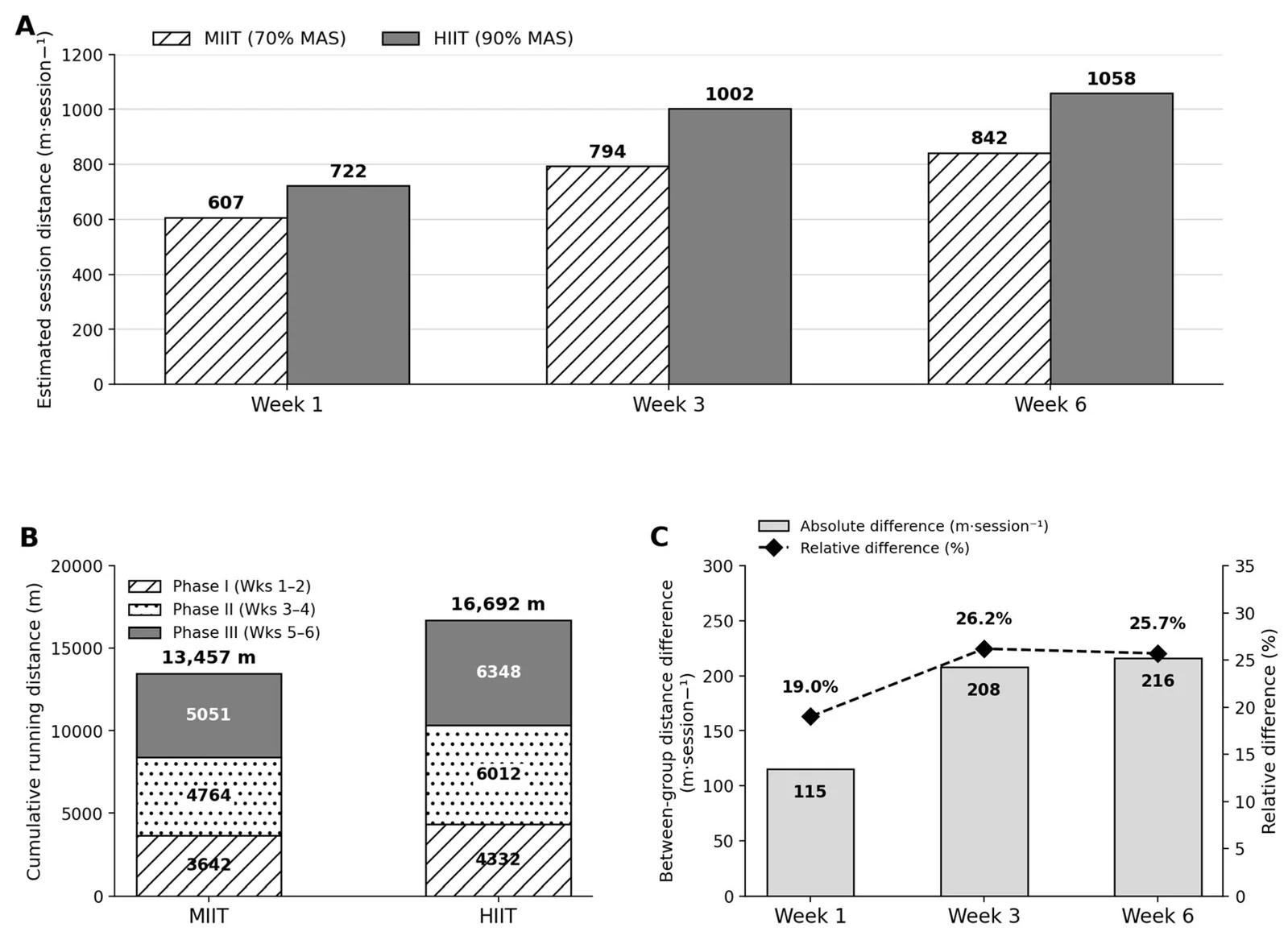
Estimated running workload across the 6-week interval-training intervention. (**A**) Estimated session running distance at Weeks 1, 3, and 6 for moderate-intensity interval training (MIIT; 70% MAS) and high-intensity interval training (HIIT; 90% MAS). (**B**) Phase-specific cumulative running distance across three consecutive two-week training phases: Phase I, Weeks 1–2; Phase II, Weeks 3–4; and Phase III, Weeks 5–6. Total cumulative running distance across 18 sessions was 16,692 m for HIIT and 13,457 m for MIIT. (**C**) Absolute and relative between-group differences in estimated session running distance at each time point. Relative differences were calculated as [(HIIT − MIIT)/MIIT] × 100. Estimates were derived from group-mean MAS values. MAS, maximal aerobic speed.

Although session duration (50 min), interval configuration (1:1 work-to-recovery ratio), treadmill incline (0°), and training frequency (3 sessions·week^−1^) were identical between groups, total external workload differed because running intensity was prescribed relative to each animal’s MAS. Estimated running distance per session was derived from the interval structure, including warm-up and cool-down components, using the following expressions: MIIT = 50 + 24 × MAS and HIIT = 50 + 28 × MAS (m·session^−1^), where MAS is expressed in m·min^−1^. The coefficients reflect the combined distance covered during work and active-recovery intervals at 70%/50% MAS (MIIT) and 90%/50% MAS (HIIT), respectively. Session-distance estimates increased progressively over time in both groups (Figure 4A). Phase-specific cumulative running distances showed that total distance across the six-week intervention was 13,457 m for MIIT and 16,692 m for HIIT (Figure 4B). The relative between-group difference in estimated session distance ranged from 19.0% at Week 1 to 26.2% at Week 3 and 25.7% at Week 6 (Figure 4C). Taken together, these values confirm that the protocols were not work-matched. Thus, the stronger insulin-resistance response observed under HIIT should be interpreted as the combined influence of higher relative intensity and greater cumulative workload, not intensity alone.

### 3.4. Integrated Molecular and Metabolic Disruptions in STZ–NA-Induced Diabetes

Residual diagnostics did not reveal major departures from normality or variance homogeneity; however, with *n* = 5 per group, Shapiro–Wilk and Levene tests were interpreted cautiously because of their limited power. One-way ANOVA demonstrated significant between-group effects across the molecular and metabolic variables evaluated, as summarized in Table 3. Post hoc pairwise comparisons were subsequently examined using Tukey’s honestly significant difference (HSD) test, and all gene-expression outcomes remained significant after Benjamini–Hochberg false discovery rate correction.

Relative to healthy controls (HC), STZ–NA induction produced marked suppression of skeletal muscle myokine and mitochondrial biogenesis-related gene expression in the diabetic control (DC) group (Table 3). The largest diabetes-associated reductions were observed for FNDC5 and OSTN, indicating pronounced impairment of exercise-responsive myokine signaling. Similarly, PGC-1α, TFAM, CCO, and UCP3 were consistently lower in DC than in HC, supporting a coordinated downregulation of mitochondrial transcriptional regulation in diabetic skeletal muscle. Tukey-adjusted comparisons confirmed significant HC–DC differences for all six transcriptional targets, with generally large to very large standardized effects (Table 4).

In parallel, DC animals exhibited severe systemic metabolic derangement (Table 3). Compared with HC, fasting insulin, fasting glucose, and HOMA-IR were all markedly elevated, whereas QUICKI and HOMA-β were significantly reduced, indicating impaired insulin sensitivity and reduced β-cell functional index. The TyG index was also significantly higher in DC than in HC, consistent with combined hepatic and peripheral insulin resistance. In the lipid profile, total cholesterol was markedly elevated in DC, whereas triglycerides showed only a modest overall group effect and no clear HC–DC separation in Tukey-adjusted analysis (Table 4).

Taken together, these data confirm that STZ–NA induction produced a robust diabetic phenotype characterized by concurrent disruption of skeletal muscle transcriptional homeostasis and systemic metabolic regulation (Table 3 and Table 4). This integrated molecular–metabolic impairment provides the biological context for interpreting the subsequent training responses.

### 3.5. Effects of Interval Training on Myokine Gene Expression

Compared with DC, FNDC5 expression was significantly higher in both MIIT (*p* = 0.022) and HIIT (*p* < 0.001). OSTN showed a more selective pattern, as follows: expression was significantly higher in HIIT than DC (*p* = 0.007), whereas the increase in MIIT did not reach Tukey-adjusted significance (*p* = 0.107). The MIIT-versus-HIIT comparisons were not statistically significant for either FNDC5 or OSTN, indicating that the present sample did not provide definitive evidence for distinct myokine transcript responses between the two interval-training protocols.

**Figure 5:**
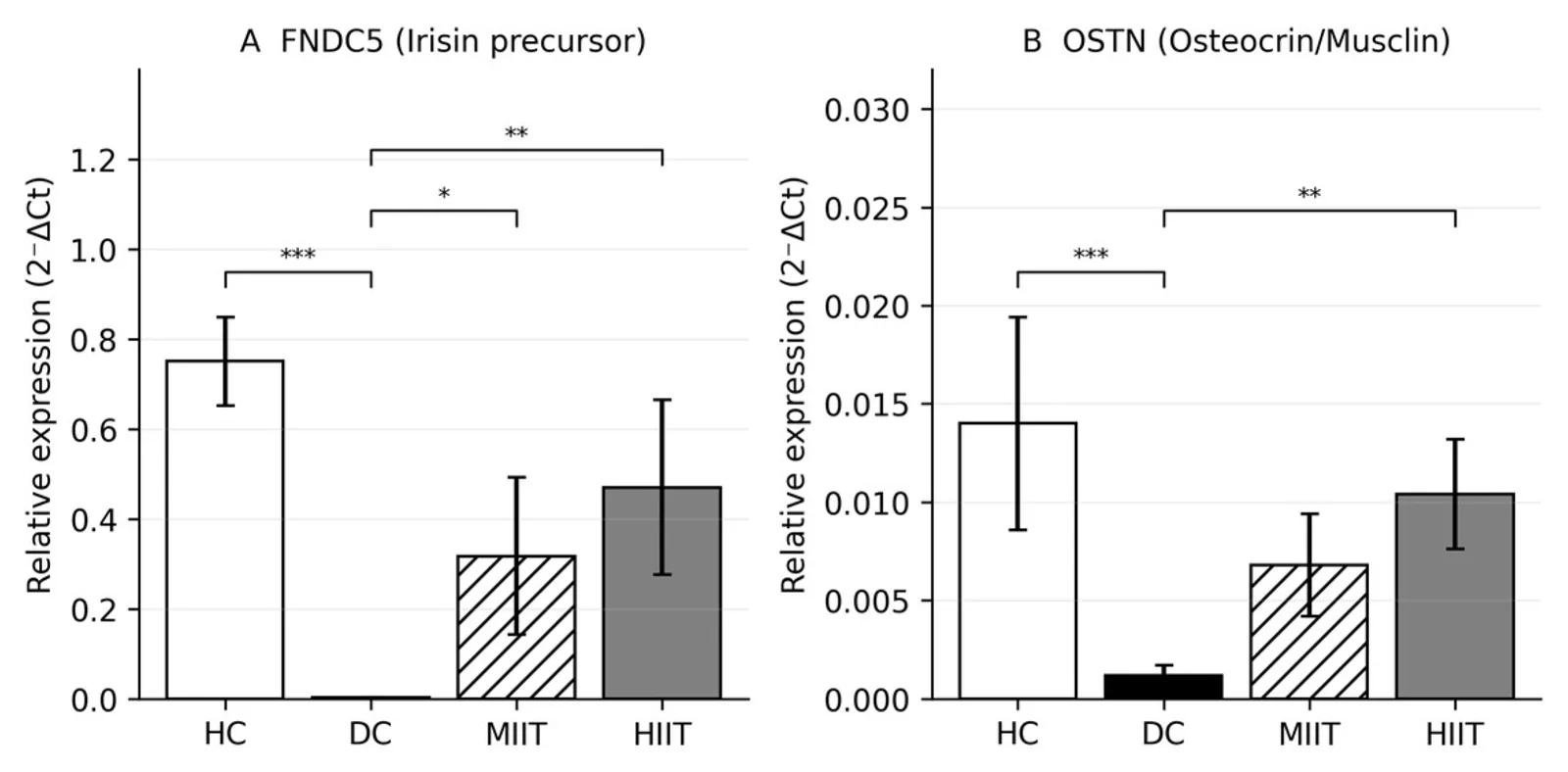
Skeletal muscle myokine expression after 6 weeks of interval training in STZ–NA diabetic rats. Relative mRNA levels of (**A**) FNDC5 and (**B**) OSTN are shown as 2^−ΔCt^ values. FNDC5 was significantly higher in both MIIT and HIIT versus DC, whereas OSTN reached significance only in HIIT versus DC. No significant MIIT–HIIT differences were observed. Data are mean ± SD (*n* = 5/group). * *p* < 0.05, ** *p* < 0.01, *** *p* < 0.001. (Note: group labels (HC = Healthy control, DC = Diabetic control, MIIT = Moderate-intensity interval training, HIIT = High-intensity interval training).

Overall, these findings indicate that interval training partially attenuated diabetes-associated suppression of exercise-responsive myokine expression. HIIT showed a consistently stronger numerical response, but the absence of significant MIIT–HIIT contrasts means that protocol-specific superiority at the transcript level should not be inferred (Table 3 and Table 4; Figure 5).

### 3.6. Effects of Interval Training on Mitochondrial Biogenesis Markers

**Figure 6:**
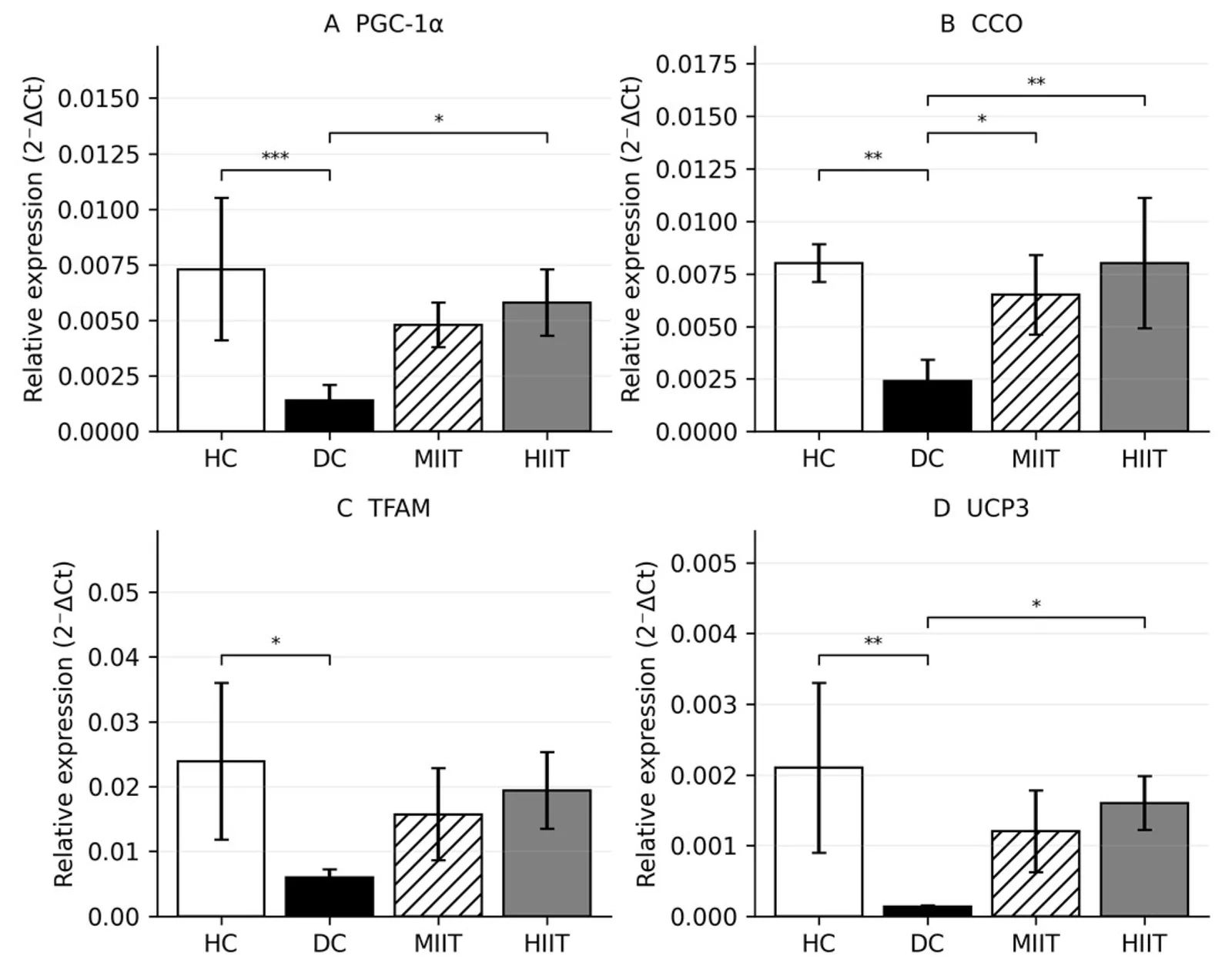
Skeletal muscle mitochondrial gene expression following interval training in STZ–NA diabetic rats. Relative mRNA levels of (**A**) PGC-1α, (**B**) CCO, (**C**) TFAM, and (**D**) UCP3 are shown as 2^−ΔCt^ values. Compared with DC, HIIT significantly increased PGC-1α, CCO, and UCP3; MIIT reached significance only for CCO. TFAM did not show a significant DC-versus-training post hoc difference. No significant MIIT–HIIT differences were observed. Data are mean ± SD (*n* = 5/group). * *p* < 0.05, ** *p* < 0.01, *** *p* < 0.001.

Compared with DC, interval training increased several mitochondrial biogenesis-related transcripts, but the statistical pattern differed by gene. HIIT significantly increased PGC-1α (Figure 6A), CCO, and UCP3 relative to DC, whereas MIIT reached significance only for CCO. TFAM showed a moderate overall group effect but no significant Tukey-adjusted DC-versus-training comparison. No MIIT–HIIT contrast was statistically significant for the mitochondrial gene-expression outcomes.

For CCO, both training groups showed significant recovery relative to DC, with significant DC vs. MIIT (*p* = 0.021) and DC vs. HIIT (*p* = 0.002) differences and very large effect sizes (Table 4; Figure 6B). Neither MIIT nor HIIT differed significantly from HC, and the MIIT–HIIT contrast remained non-significant (*p* = 0.627). This pattern indicates substantial restoration of this oxidative marker irrespective of training intensity.

For TFAM, the overall ANOVA effect was more moderate, and post hoc results were less robust (Table 3). The HC–DC contrast remained significant (*p* = 0.010), confirming diabetes-associated suppression, but neither DC vs. MIIT (*p* = 0.235) nor DC vs. HIIT (*p* = 0.057) reached Tukey-adjusted significance, although the latter approached the threshold and was associated with a very large effect size (Table 4; Figure 6C). Accordingly, TFAM should be interpreted more cautiously than the other mitochondrial markers, as the absence of significance may reflect limited power rather than absence of biological responsiveness.

For UCP3, HIIT significantly increased expression relative to DC (*p* = 0.022), whereas MIIT did not (*p* = 0.109) (Table 4; Figure 6D). As with the other markers, neither trained group differed significantly from HC, and no significant MIIT–HIIT difference was detected (*p* = 0.815). Collectively, these findings indicate partial improvement in mitochondrial transcriptional status in diabetic skeletal muscle, with the clearest post hoc support for CCO and selective HIIT-related increases in PGC-1α and UCP3, while TFAM should be interpreted cautiously (Table 3 and Table 4; Figure 6).

### 3.7. Effects of Interval Training on Insulin Resistance, β-Cell Function, and Metabolic Indices

**Figure 7:**
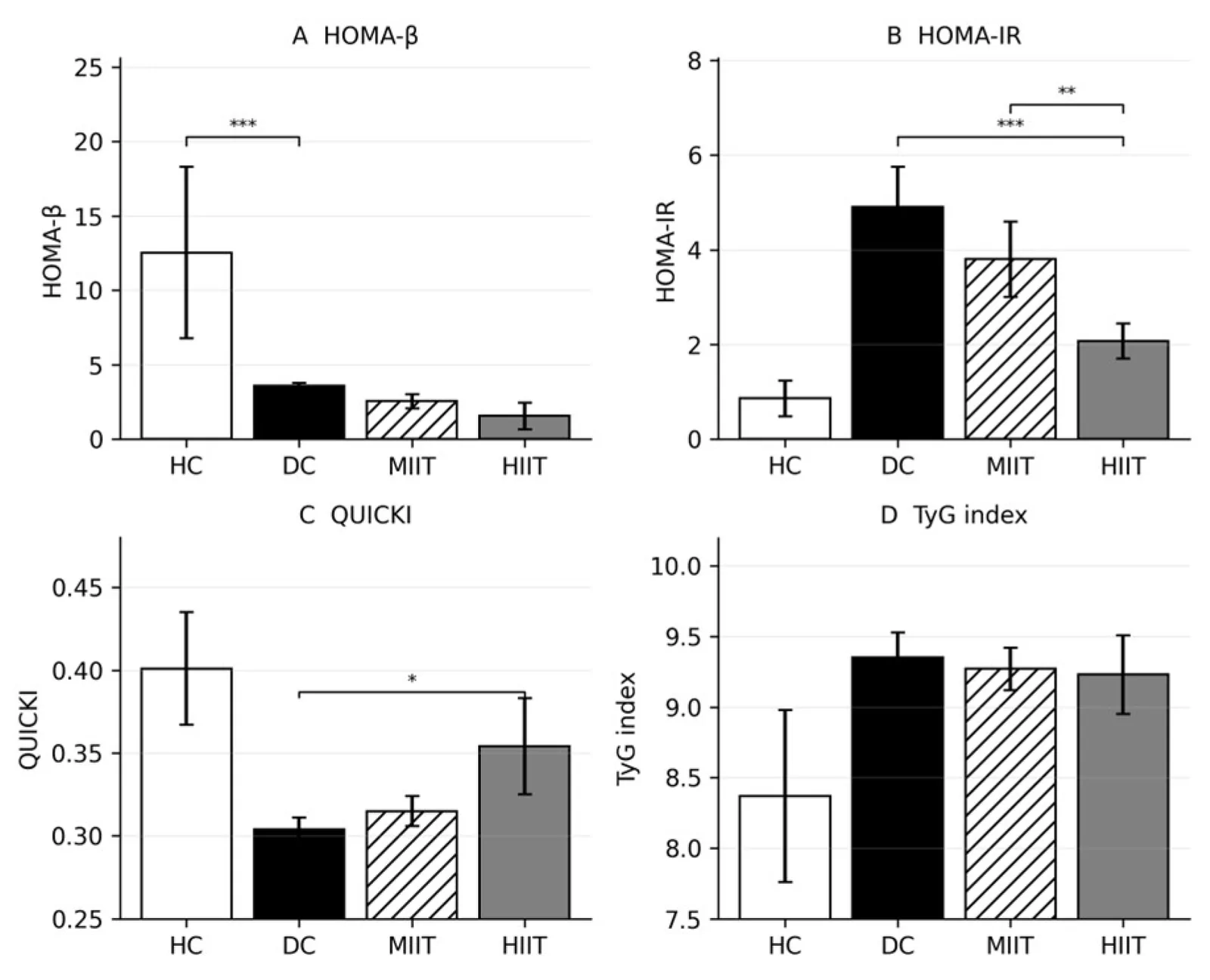
Metabolic indices following 6 weeks of interval training in STZ–NA diabetic rats. (**A**) HOMA-β, (**B**) HOMA-IR, (**C**) QUICKI, and (**D**) TyG index. HOMA-IR was significantly reduced in HIIT versus DC and MIIT, whereas MIIT did not differ from DC. QUICKI improved significantly only in HIIT versus DC. HOMA-β and TyG did not differ significantly among diabetic groups. Data are mean ± SD (*n* = 5/group). * *p* < 0.05, ** *p* < 0.01, *** *p* < 0.001.

HIIT was associated with lower fasting insulin and HOMA-IR than both DC and MIIT, whereas MIIT did not significantly reduce HOMA-IR relative to DC. QUICKI was significantly higher in HIIT than DC, indicating improved insulin sensitivity, but the MIIT-versus-HIIT difference did not reach significance for QUICKI. HOMA-β and TyG did not differ significantly among the diabetic groups, and fasting glucose remained markedly elevated in all diabetic animals. These findings support improvement in selected insulin-related indices rather than complete restoration of glycaemic control (Table 3 and Table 4; Figure 7).

### 3.8. Exploratory Correlations Between Skeletal Muscle Gene Expression and Systemic Metabolic Indices

**Figure 8:**
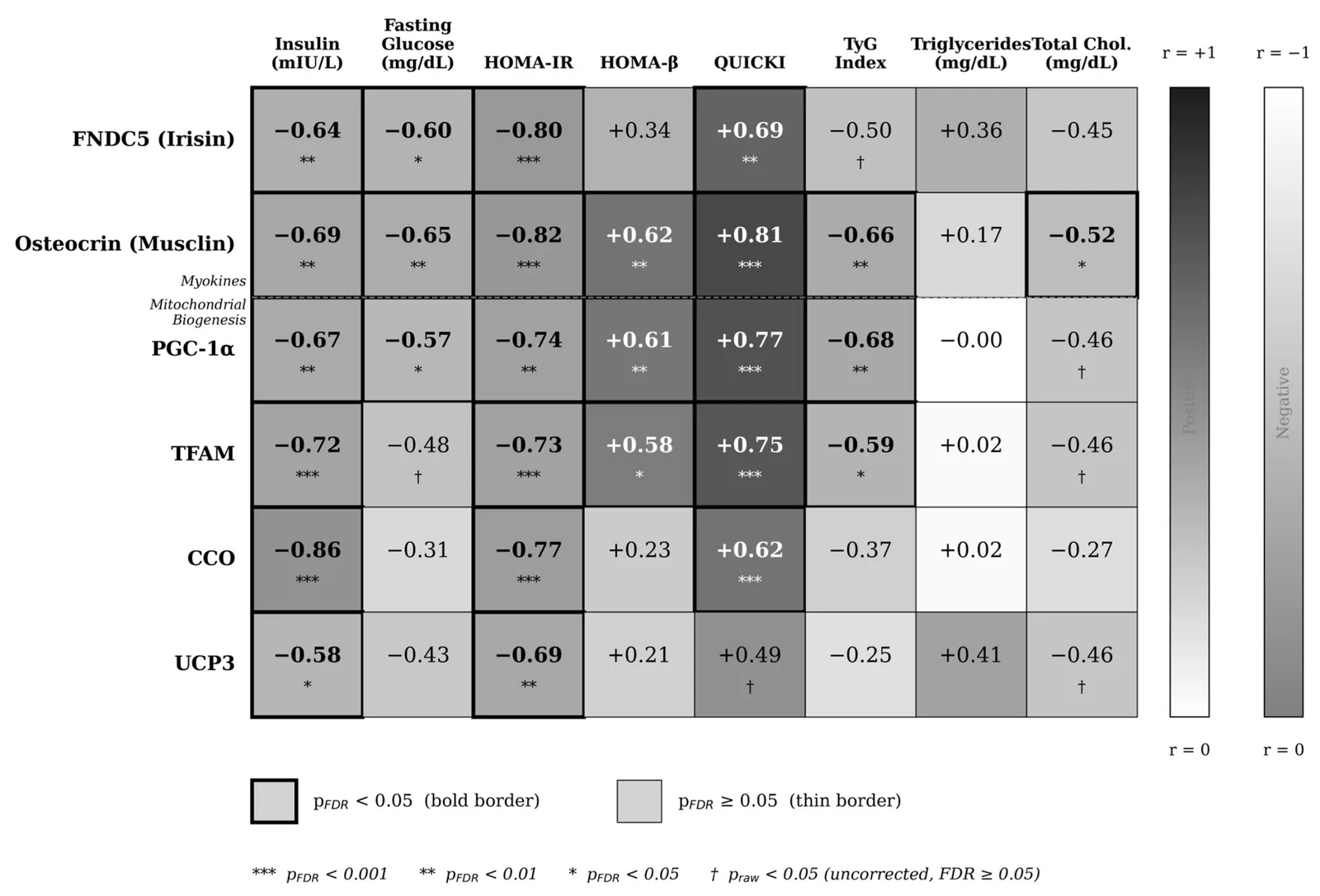
Pearson correlation matrix between skeletal muscle gene expression and metabolic indices. Heatmap of correlation coefficients (r) across all animals (*n* = 20; *n* = 18 for FNDC5). Color indicates direction and magnitude of association. Bold borders denote FDR-adjusted significance (q < 0.05). * *p* < 0.05, ** *p* < 0.01, *** *p* < 0.001.

Exploratory correlation analyses suggested associations between several skeletal muscle gene-expression variables and insulin-related metabolic indices (Figure 8). Because these analyses were performed in a small sample and across pooled groups, they should be interpreted as hypothesis-generating and associative rather than mechanistic or causal. 

The strongest individual association was observed between CCO expression and fasting insulin, while osteocrin showed the broadest overall correlation profile. FNDC5, PGC-1α, and TFAM also demonstrated robust associations with insulin resistance and insulin sensitivity indices, whereas UCP3 showed weaker but directionally concordant relationships.

Taken together, these results support the presence of a coordinated link between skeletal muscle gene expression and whole-body metabolic regulation in the STZ–NA model. Nevertheless, the observed correlations do not establish causality and should be interpreted as evidence of biological coupling rather than direct mechanistic mediation.

### 3.9. Fold-Change Expression and Effect-Size Summary

**Figure 9:**
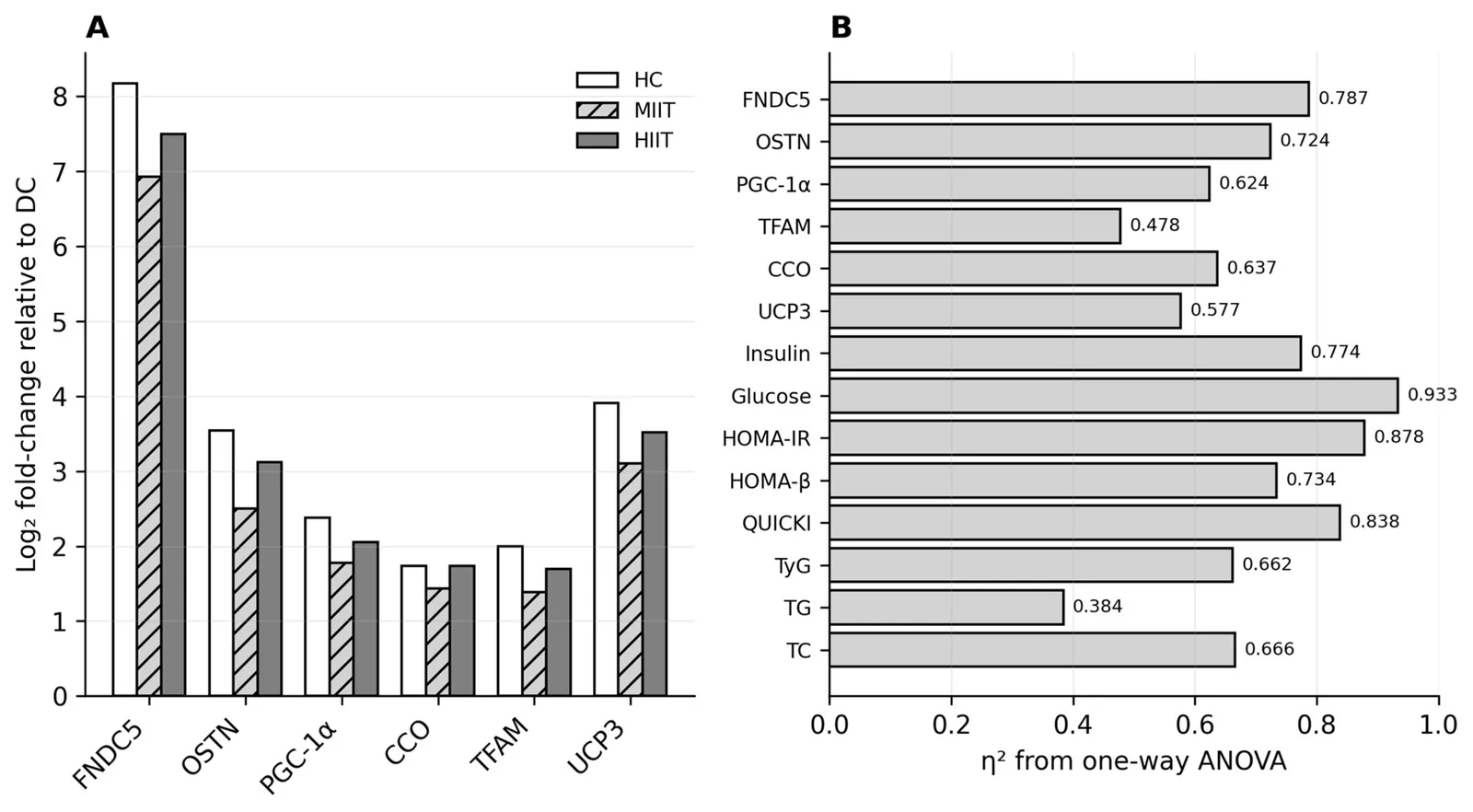
Fold-change expression and effect sizes across outcomes. (**A**) Log_2_ fold-change relative to DC for gene-expression outcomes. (**B**) Effect sizes (η^2^) from one-way ANOVA. HIIT showed greater numerical increases than MIIT for several genes; however, most MIIT–HIIT contrasts were not statistically significant. Panel A presents descriptive point estimates calculated from group mean 2^−ΔCt^ values and was not used for inference. Variability is presented in Figure 5 and Figure 6, and inferential results are reported in Table 3 and Table 4.

Fold-change summaries indicated that diabetes induction produced large transcriptional suppression relative to HC, whereas both training protocols partially restored gene-expression levels compared with DC. For most molecular outcomes, the magnitude of response was numerically greater in HIIT, but direct MIIT-versus-HIIT comparisons were not statistically significant. Figure 9A is a descriptive visualization of group-level log_2_ fold-change point estimates calculated from mean 2^−ΔCt^ values relative to DC; it was not used for inferential testing. Error bars are therefore not displayed in this panel. Variability and inferential statistics are reported in Figure 5 and Figure 6 and Table 3 and Table 4.

STZ–NA induction produced a profound suppression of myokine expression, most prominently for FNDC5, which showed an extreme downregulation in DC relative to HC. Both MIIT and HIIT elicited substantial restoration of FNDC5 expression, with a consistent directional advantage for HIIT, although recovery remained incomplete relative to healthy baseline (Figure 9A). A similar but less pronounced pattern was observed for UCP3 and osteocrin, indicating partial normalization of mitochondrial and myokine signaling.

In contrast, mitochondrial biogenesis regulators (PGC-1α, TFAM, CCO) exhibited more moderate fold-change responses, reflecting both a lower degree of diabetes-induced suppression and a comparatively constrained responsiveness to training. Collectively, these patterns indicate that myokines represent the most dynamically regulated transcriptional axis in response to both diabetic insult and exercise intervention (Figure 9A).

Effect-size analysis corroborated these observations (Figure 9B). All outcomes demonstrated large-to-very-large between-group effects (η^2^ range: 0.384–0.933), with the greatest magnitudes observed for fasting glucose, HOMA-IR, QUICKI, and FNDC5. These findings indicate that glycaemic dysregulation, insulin-resistance indices, and myokine suppression accounted for a large proportion of between-group variance.

Given the modest sample size (*n* = 5 per group), statistical power was sufficient for detecting large effects but limited for resolving smaller between-protocol differences. Accordingly, non-significant MIIT–HIIT contrasts should be interpreted as limited evidence for protocol-specific separation rather than proof of equivalence. Overall, the combined fold-change and effect-size profiles support a robust but partially restored transcriptional response in trained diabetic skeletal muscle (Figure 9).

## 4. Discussion

The present study provides integrated evidence that six weeks of interval training partially attenuated the molecular and metabolic disturbances induced by STZ–NA-mediated type 2 diabetes in rat skeletal muscle. Diabetes is characterized by marked suppression of skeletal muscle myokine- and mitochondrial biogenesis-related transcripts and by deterioration in insulin-related metabolic indices. Both MIIT and HIIT partially restored FNDC5, OSTN, PGC-1α, TFAM, CCO, and UCP3 expression compared with diabetic controls; however, most molecular outcomes did not differ significantly between the two training protocols. Functionally, both protocols improved aerobic capacity, whereas HIIT produced lower fasting insulin and HOMA-IR than MIIT under the present non-work-matched conditions.

The diabetic control group displayed lower expression of FNDC5, OSTN, PGC-1α, TFAM, CCO, and UCP3 than healthy controls, supporting a coordinated impairment of skeletal muscle transcriptional regulation in this experimental model. This pattern agrees with literature describing mitochondrial dysfunction and disturbed myokine signaling in type 2 diabetic skeletal muscle [8,9,34,35]. It extends those observations by assessing two exercise-responsive myokine transcripts together with four mitochondrial-related transcripts in the same experimental cohort. Because the present study quantified mRNA abundance only, the findings represent transcriptional adaptations and cannot be taken as direct evidence of altered protein abundance, enzyme activity, mitochondrial respiration, or myokine secretion.

Exercise-induced mitochondrial adaptation is commonly linked to transcriptional networks involving PGC-1α and downstream mitochondrial regulators. Workload-matched interval training has increased mitochondrial-biogenesis regulators in diabetic rat soleus [14], while other diabetic rodent studies have reported HIIT-related improvements in skeletal-muscle metabolic pathways [15,16]. In db/db mice and adults with type 2 diabetes, interval training has also improved skeletal-muscle mitochondrial function or capacity [36,37]. The partial restoration of PGC-1α, CCO, UCP3, and possibly TFAM in the present study is directionally consistent with these reports, but the response was marker-specific and less complete than normalization to healthy control levels. Differences in diabetes model, muscle phenotype, intervention dose, and molecular endpoint limit direct quantitative comparison. Moreover, the absence of protein-level and functional mitochondrial measurements prevents firm mechanistic conclusions.

The myokine-related findings are also biologically relevant. FNDC5 and OSTN were strongly suppressed in diabetic controls, suggesting impaired exercise-responsive signaling in skeletal muscle. The FNDC5 response is consistent with the experimentally described PGC-1α–FNDC5 axis [18] and with observations linking muscle FNDC5 expression to diabetic metabolic status [19,20,38]. OSTN/musclin is an activity-responsive myokine associated with endurance adaptation [39], and experimental evidence suggests potential protective relevance in diabetes-related pathology [40]. In the present study, FNDC5 was significantly higher in both trained groups than in diabetic controls, whereas OSTN reached statistical significance only in HIIT; however, MIIT–HIIT contrasts were not significant. Because circulating irisin or osteocrin and protein abundance were not measured, higher transcript levels cannot be assumed to indicate increased secretion or endocrine activity.

The metabolic results suggest that training-related benefits were more apparent for insulin-related indices than for fasting glucose. Improvements in insulin resistance and muscle metabolic capacity after interval training have also been reported in diabetic rodent models [15,16] and in studies of db/db mice and adults with type 2 diabetes [36,37]. In the present study, HIIT reduced fasting insulin and HOMA-IR and increased QUICKI relative to diabetic controls, whereas fasting glucose remained markedly elevated across all diabetic groups. This pattern indicates improved surrogate insulin sensitivity without glycaemic normalization. The persistent hyperglycaemia may reflect the severity of STZ–NA-induced β-cell impairment, the six-week intervention duration, and the inability of fasting surrogate indices to capture dynamic glucose disposal.

A central interpretive issue is that the protocols were not work-matched. Unlike the workload-matched diabetic-rat study by Delfan et al. [14], the present HIIT group completed approximately 24.0% more estimated cumulative running distance than MIIT. Comparisons with work-matched evidence [14] and conceptual intensity comparisons [17] must therefore remain qualitative rather than being interpreted as a direct test of intensity alone. Consequently, the superior HOMA-IR response in HIIT may reflect the combined influence of higher relative intensity and greater mechanical workload. Future work-matched designs are required to isolate the independent contribution of exercise intensity.

Exploratory correlations suggested coordinated links between skeletal muscle transcripts and systemic insulin-related indices. The direction of these associations is compatible with proposed myokine–metabolic cross-talk [34,35,38,39,40]. However, correlations calculated across pooled groups may be influenced by between-group separation, and associations between mRNA and metabolic indices do not demonstrate myokine secretion, mitochondrial function, or causal mediation.

Several limitations should be acknowledged. First, the final sample size was small, limiting power to detect small-to-moderate between-protocol differences. Although the balanced design and residual diagnostics supported the planned parametric analyses, formal assumption tests have low sensitivity at *n* = 5 per group; effect sizes and confidence intervals should therefore be considered alongside *p* values. Second, the HIIT and MIIT protocols were not matched for cumulative workload, preventing attribution of between-protocol differences to intensity alone. Third, only male rats were studied, limiting generalizability to females. Fourth, molecular analyses were limited to mRNA expression; protein abundance, myokine secretion, enzyme activity, mitochondrial respiration, and histological outcomes were not assessed. Fifth, MAS was determined from treadmill performance without VO_2_ or CO_2_ measurement and should not be interpreted as gas-exchange-confirmed vVO_2_max. Sixth, only the gastrocnemius was sampled; responses in predominantly slow-oxidative muscles such as the soleus may differ. Finally, correlation analyses were exploratory and should be interpreted as hypothesis-generating rather than mechanistic.

## 5. Conclusions

Six weeks of interval training partially attenuated diabetes-associated impairments in skeletal muscle myokine- and mitochondrial biogenesis-related gene expression and improved selected insulin-related metabolic indices in STZ–NA-induced diabetic rats. Most molecular adaptations were broadly comparable between MIIT and HIIT, whereas HIIT was associated with lower fasting insulin and HOMA-IR than MIIT under non-work-matched conditions. Because HIIT produced greater cumulative external workload, these findings should be interpreted as reflecting the combined influence of higher relative intensity and greater training load rather than intensity alone. The molecular findings are transcriptional and do not demonstrate increased protein abundance, myokine secretion, or mitochondrial function. Future studies using work-matched designs, larger samples, both sexes, multiple muscle phenotypes, and protein-level or functional mitochondrial measurements are needed to clarify the mechanisms underlying interval-training adaptations in diabetic skeletal muscle.

## Figures and Tables

**Table 1:** Characteristics of the Interval Training Protocols.

Parameter	Control	MIIT	HIIT
Animals (*n*)	5	5	5
Session duration (min)	50	50	50
Training frequency (sessions·week^−1^)	3	3	3
Intervention duration (weeks)	6	6	6
Warm-up	No running	5 min @ 5 m·min^−1^	5 min @ 5 m·min^−1^
Interval work intensity	—	70% MAS	90% MAS
Active recovery intensity	—	50% MAS	50% MAS
Work:recovery ratio	—	1:1 (1 min:1 min)	1:1 (1 min:1 min)
Number of cycles	—	20	20
Total interval duration (min)	—	40	40
Cool-down	No running	5 min @ 5 m·min^−1^	5 min @ 5 m·min^−1^
Treadmill incline	Level (0°)	Level (0°)	Level (0°)
MAS assessment	—	Weeks 1, 3, 6	Weeks 1, 3, 6
Estimated session distance	—	50 + 24 × MAS	50 + 28 × MAS
Estimated 6-week distance	—	13,457 m	16,692 m

**Table 2:** Primer sequences used for RT-qPCR analysis.

Gene	RefSeq Accession	Forward Primer (5′→3′)	Reverse Primer (5′→3′)	Amplicon Size (bp)
FNDC5	NM_001270981.4	CAGCCATTGTCACTGGCCTG	GGGAGAGAGAGAGGGAGAAGGAG	142
OSTN	NM_001106720.1	GATGTGGGGCTCAGGAAAGG	GAGAGTGTGTCAAGGGGAGA	136
CCO	NM_017202	TTTGGGGGCAAGGAAAGAGT	ATACATGGCAGAAGTGGGAGA	128
UCP3	NM_013167.2	GCAGCCTGTTTTGCTGCTCT	GGTTCTCCCCTTGGATGTG	131
PGC-1α	NM_031347.1	AACAAGCACTTCGGTCATCC	CTTCGCTGTCATCAAACAGG	145
TFAM	NM_031326.2	CGA GGT CTT TTT GGT TTT CC	AAG GTG TAT GAA GCG GAT TTT	269
GAPDH	NM_017008.4	CATACTCAGCACCAGCATCACC	AAGTTCAACGGCACAGTCAAGG	121

**Table 3:** Group means, dispersion measures, and one-way ANOVA results for skeletal muscle gene-expression and metabolic outcomes. Gene-expression statistics were analyzed using ΔCt values, whereas 2^−ΔCt^ values are presented for interpretability.

Variable	η^2^	*p* ^c^	HC (*n* = 5)	DC (*n* = 5)	MIIT (*n* = 5)	HIIT (*n* = 5)
FNDC5	0.787	<0.001	0.750 ± 0.098	0.0026 ± 0.0005	0.317 ± 0.175	0.470 ± 0.194
OSTN	0.724	<0.001	0.0140 ± 0.0054	0.0012 ± 0.0005	0.0068 ± 0.0026	0.0104 ± 0.0028
PGC-1α	0.624	<0.001	0.0073 ± 0.0032	0.0014 ± 0.0007	0.0048 ± 0.0010	0.0058 ± 0.0015
TFAM	0.478	0.013	0.0239 ± 0.0121	0.0060 ± 0.0012	0.0157 ± 0.0071	0.0194 ± 0.0059
CCO	0.637	<0.001	0.0080 ± 0.0009	0.0024 ± 0.0010	0.0065 ± 0.0019	0.0080 ± 0.0031
UCP3	0.577	0.003	0.0021 ± 0.0012	0.00014 ± 0.00001	0.0012 ± 0.00058	0.0016 ± 0.00038
Insulin (mIU/L)	0.774	<0.001	2.346 ± 0.452	4.120 ± 0.420	3.080 ± 0.590	1.820 ± 0.580
Glucose (mg/dL)	0.933	<0.001	144.2 ± 40.2	478.6 ± 37.3	499.2 ± 26.6	474.8 ± 64.0
HOMA-IR	0.878	<0.001	0.86 ± 0.38	4.90 ± 0.85	3.80 ± 0.80	2.07 ± 0.37
HOMA-β	0.734	<0.001	12.52 ± 5.76	3.58 ± 0.20	2.55 ± 0.48	1.56 ± 0.90
QUICKI	0.838	<0.001	0.401 ± 0.034	0.304 ± 0.007	0.315 ± 0.009	0.354 ± 0.029
TyG Index	0.662	<0.001	8.37 ± 0.61	9.35 ± 0.18	9.27 ± 0.15	9.23 ± 0.28
Triglycerides (mg/dL)	0.384	0.046	64.6 ± 20.2	48.8 ± 10.0	41.2 ± 6.3	45.7 ± 8.6
Total Cholesterol (mg/dL)	0.666	<0.001	39.2 ± 7.5	106.0 ± 5.4	83.3 ± 19.0	102.3 ± 36.5

Data are presented as mean ± SD. Group differences were evaluated using one-way ANOVA. Gene-expression values are presented as relative normalized expression units (2^−ΔCt^). Statistical analyses for qPCR outcomes were performed on ΔCt values. Metabolic outcomes include fasting insulin, fasting glucose, HOMA-IR, HOMA-β, QUICKI, TyG index, triglycerides, and total cholesterol. HC, healthy control; DC, diabetic control; MIIT, moderate-intensity interval training; HIIT, high-intensity interval training; η^2^, eta squared; HOMA-IR, homeostatic model assessment of insulin resistance; HOMA-β, homeostatic model assessment of β-cell function; QUICKI, quantitative insulin sensitivity check index; TyG, triglyceride–glucose index.

**Table 4:** Tukey-adjusted post hoc pairwise comparisons and standardized effect sizes for molecular and metabolic outcomes.

Comparison	Mean Difference	95% CI [Lower, Upper]	*p* Tukey	Sig.	Hedges’ g
FNDC5—F = 17.228, *p* < 0.001, η^2^ = 0.787 (unit: 2^−ΔCt^)					
HC vs. DC	−0.7392	[−1.0546, −0.4238]	<0.001	***	+8.390
HC vs. MIIT	−0.4246	[−0.7400, −0.1092]	0.007	**	+2.272
HC vs. HIIT	−0.2714	[−0.5868, +0.0439]	0.103	ns	+1.341
DC vs. MIIT	+0.3146	[+0.0415, +0.5877]	0.022	*	−2.290
DC vs. HIIT	+0.4678	[+0.1946, +0.7409]	<0.001	**	−3.079
MIIT vs. HIIT	+0.1532	[−0.1200, +0.4263]	0.394	ns	−0.748
OSTN—F = 13.960, *p* < 0.001, η^2^ = 0.724 (unit: 2^−ΔCt^)					
HC vs. DC	−0.01285	[−0.01950, −0.00720]	<0.001	***	+3.096
HC vs. MIIT	−0.00719	[−0.01384, −0.00054]	0.030	*	+1.601
HC vs. HIIT	−0.00374	[−0.01039, +0.00291]	0.393	ns	+0.846
DC vs. MIIT	+0.00566	[−0.00099, +0.01231]	0.107	ns	−2.655
DC vs. HIIT	+0.00911	[+0.00246, +0.01576]	0.007	**	−4.013
MIIT vs. HIIT	+0.00345	[−0.00320, +0.01010]	0.461	ns	−1.149
PGC-1α—F = 8.849, *p* < 0.001, η^2^ = 0.624 (unit: 2^−ΔCt^)					
HC vs. DC	−0.00586	[−0.00947, −0.00225]	<0.001	***	+2.284
HC vs. MIIT	−0.00234	[−0.00595, +0.00127]	0.304	ns	+0.952
HC vs. HIIT	−0.00136	[−0.00497, +0.00225]	0.735	ns	+0.557
DC vs. MIIT	+0.00352	[−0.00009, +0.00713]	0.056	ns	−3.511
DC vs. HIIT	+0.00450	[+0.00089, +0.00811]	0.013	*	−3.330
MIIT vs. HIIT	+0.00098	[−0.00263, +0.00459]	0.867	ns	−0.684
TFAM—F = 4.887, *p* = 0.013, η^2^ = 0.478 (unit: 2^−ΔCt^)					
HC vs. DC	−0.01796	[−0.03178, −0.00414]	0.010	*	+1.863
HC vs. MIIT	−0.00825	[−0.02207, +0.00557]	0.370	ns	+0.740
HC vs. HIIT	−0.00450	[−0.01832, +0.00932]	0.800	ns	+0.419
DC vs. MIIT	+0.00971	[−0.00411, +0.02353]	0.235	ns	−1.702
DC vs. HIIT	+0.01346	[−0.00036, +0.02728]	0.057	ns	−2.818
MIIT vs. HIIT	+0.00375	[−0.01007, +0.01757]	0.871	ns	−0.513
CCO (Cytochrome c Oxidase)—F = 9.368, *p* < 0.001, η^2^ = 0.637 (unit: 2^−ΔCt^)					
HC vs. DC	−0.00563	[−0.00920, −0.00206]	0.002	**	+5.052
HC vs. MIIT	−0.00146	[−0.00503, +0.00211]	0.660	ns	+0.875
HC vs. HIIT	+0.00008	[−0.00349, +0.00365]	1.000	ns	−0.023
DC vs. MIIT	+0.00417	[+0.00060, +0.00774]	0.021	*	−2.453
DC vs. HIIT	+0.00571	[+0.00214, +0.00928]	0.002	**	−2.221
MIIT vs. HIIT	+0.00154	[−0.00203, +0.00511]	0.627	ns	−0.534
UCP3 (Uncoupling Protein 3)—F = 7.287, *p* = 0.003, η^2^ = 0.577 (unit: 2^−ΔCt^)					
HC vs. DC	−0.002036	[−0.003293, −0.000779]	0.002	**	+2.096
HC vs. MIIT	−0.000969	[−0.002226, +0.000288]	0.175	ns	+0.903
HC vs. HIIT	−0.000570	[−0.001827, +0.000687]	0.586	ns	+0.560
DC vs. MIIT	+0.001067	[−0.000190, +0.002324]	0.109	ns	−2.336
DC vs. HIIT	+0.001466	[+0.000209, +0.002723]	0.022	*	−4.895
MIIT vs. HIIT	+0.000399	[−0.000858, +0.001656]	0.815	ns	−0.732
Insulin (mIU/L)—F = 18.316, *p* < 0.001, η^2^ = 0.774 (unit: mIU/L)					
HC vs. DC	+1.781	[+0.838, +2.725]	<0.001	***	−3.647
HC vs. MIIT	+0.736	[−0.207, +1.679]	0.157	ns	−1.260
HC vs. HIIT	−0.520	[−1.464, +0.423]	0.418	ns	+0.895
DC vs. MIIT	−1.045	[−1.989, −0.102]	0.027	*	+1.823
DC vs. HIIT	−2.302	[−3.245, −1.358]	<0.001	***	+4.035
MIIT vs. HIIT	−1.256	[−2.200, −0.313]	0.008	**	+1.920
Fasting Glucose (mg/dL)—F = 74.284, *p* < 0.001, η^2^ = 0.933 (unit: mg/dL)					
HC vs. DC	+334.4	[+254.4, +414.4]	<0.001	***	−7.789
HC vs. MIIT	+355.0	[+275.0, +434.9]	<0.001	***	−9.403
HC vs. HIIT	+330.6	[+250.6, +410.6]	<0.001	***	−5.587
DC vs. MIIT	+20.6	[−59.4, +100.6]	0.881	ns	−0.574
DC vs. HIIT	−3.800	[−83.8, +76.2]	0.999	ns	+0.066
MIIT vs. HIIT	−24.4	[−104.4, +55.6]	0.819	ns	+0.450
HOMA-IR—F = 38.545, *p* < 0.001, η^2^ = 0.878 (unit: a.u.)					
HC vs. DC	+4.036	[+2.868, +5.204]	<0.001	***	−5.499
HC vs. MIIT	+2.936	[+1.768, +4.104]	<0.001	***	−4.201
HC vs. HIIT	+1.208	[+0.040, +2.376]	0.042	*	−2.894
DC vs. MIIT	−1.100	[−2.268, +0.068]	0.069	ns	+1.194
DC vs. HIIT	−2.828	[−3.997, −1.660]	<0.001	***	+3.872
MIIT vs. HIIT	−1.728	[−2.897, −0.560]	0.003	**	+2.486
HOMA-β—F = 14.684, *p* < 0.001, η^2^ = 0.734 (unit: a.u.)					
HC vs. DC	−8.946	[−14.242, −3.651]	<0.001	***	+1.984
HC vs. MIIT	−9.975	[−15.271, −4.679]	<0.001	***	+2.206
HC vs. HIIT	−10.819	[−16.115, −5.523]	<0.001	***	+2.370
DC vs. MIIT	−1.029	[−6.325, +4.267]	0.944	ns	+2.547
DC vs. HIIT	−1.873	[−7.168, +3.423]	0.745	ns	+2.527
MIIT vs. HIIT	−0.844	[−6.140, +4.452]	0.968	ns	+1.036
QUICKI—F = 27.590, *p* < 0.001, η^2^ = 0.838 (unit: score)					
HC vs. DC	−0.0971	[−0.1306, −0.0636]	<0.001	***	+3.575
HC vs. MIIT	−0.0863	[−0.1198, −0.0528]	<0.001	***	+3.125
HC vs. HIIT	−0.0582	[−0.0917, −0.0247]	<0.001	**	+2.114
DC vs. MIIT	+0.0109	[−0.0226, +0.0444]	0.790	ns	−1.208
DC vs. HIIT	+0.0389	[+0.0054, +0.0724]	0.020	*	−4.402
MIIT vs. HIIT	+0.0280	[−0.0055, +0.0615]	0.119	ns	−2.783
TyG Index—F = 10.434, *p* < 0.001, η^2^ = 0.662 (unit: index)					
HC vs. DC	+0.976	[+0.382, +1.571]	<0.001	**	−1.980
HC vs. MIIT	+0.900	[+0.305, +1.494]	0.003	**	−1.844
HC vs. HIIT	+0.966	[+0.371, +1.560]	0.001	**	−2.006
DC vs. MIIT	−0.077	[−0.671, +0.518]	0.982	ns	+0.422
DC vs. HIIT	−0.011	[−0.605, +0.584]	1.000	ns	+0.064
MIIT vs. HIIT	+0.066	[−0.529, +0.661]	0.988	ns	−0.450
Triglycerides (mg/dL)—F = 3.330, *p* = 0.046, η^2^ = 0.384 (unit: mg/dL)					
HC vs. DC	−15.8	[−37.5, +5.9]	0.200	ns	+0.895
HC vs. MIIT	−21.8	[−43.5, −0.1]	0.049	*	+1.332
HC vs. HIIT	−16.2	[−37.9, +5.5]	0.184	ns	+0.982
DC vs. MIIT	−6.0	[−27.7, +15.7]	0.857	ns	+0.671
DC vs. HIIT	−0.4	[−22.1, +21.3]	1.000	ns	+0.044
MIIT vs. HIIT	+5.6	[−16.1, +27.3]	0.880	ns	−0.877
Total Cholesterol (mg/dL)—F = 10.637, *p* < 0.001, η^2^ = 0.666 (unit: mg/dL)					
HC vs. DC	+66.8	[+27.3, +106.3]	<0.001	***	−9.244
HC vs. MIIT	+47.8	[+8.3, +87.3]	0.015	*	−3.033
HC vs. HIIT	+68.2	[+28.7, +107.7]	<0.001	***	−2.228
DC vs. MIIT	−19.0	[−58.5, +20.5]	0.531	ns	+1.247
DC vs. HIIT	+1.4	[−38.1, +40.9]	1.000	ns	−0.046
MIIT vs. HIIT	+20.4	[−19.1, +59.9]	0.473	ns	−0.610

Paiwise group comparisons were performed using Tukey’s honestly significant difference (HSD) test following one-way ANVA. Mean difference is expressed as the second group minus the first group for each listed comparison, with corresponding 95% confidence intervals. Standardized effects are reported as Hedges’ g with small-sample correction. Significance codes: *** *p* < 0.001, ** *p* < 0.01, * *p* < 0.05, ns, not significant. Gene-expression values are displayed as 2^−ΔCt^ for interpretability, whereas statistical comparisons were performed on ΔCt values. HC, healthy control; DC, diabetic control; MIIT, moderate-intensity interval training; HIIT, high-intensity interval training; HOMA-IR, homeostatic model assessment of insulin resistance; HOMA-β, homeostatic model assessment of β-cell function; QUICKI, quantitative insulin sensitivity check index; TyG, triglyceride–glucose index.

## Data Availability

The datasets analyzed during the present study are available from the corresponding author upon reasonable request.

## Abbreviations

UCP3	Uncoupling protein 3
TyG	Triglyceride–glucose index
TG	Triglycerides
TFAM	Mitochondrial transcription factor A
TC	Total cholesterol
T2D	Type 2 diabetes
STZ	Streptozotocin
SD	Standard deviation
RT-qPCR	Reverse transcription–quantitative polymerase chain reaction
QUICKI	Quantitative insulin sensitivity check index
PGC-1α	Peroxisome proliferator-activated receptor gamma coactivator 1-alpha
OSTN	Osteocrin/musclin
NA	Nicotinamide
MIIT	Moderate-intensity interval training
MAS	Maximal aerobic speed
HOMA-IR	Homeostatic model assessment of insulin resistance
HOMA-β	Homeostatic model assessment of β-cell function
HIIT	High-intensity interval training
HC	Healthy control
GAPDH	Glyceraldehyde-3-phosphate dehydrogenase
FNDC5	Fibronectin type III domain-containing protein 5
ELISA	Enzyme-linked immunosorbent assay
EDTA	Ethylenediaminetetraacetic acid
DC	Diabetic control
CCO	Cytochrome c oxidase
